# Network switches and their role in circadian clocks

**DOI:** 10.1016/j.jbc.2024.107220

**Published:** 2024-03-22

**Authors:** Marta del Olmo, Stefan Legewie, Michael Brunner, Thomas Höfer, Achim Kramer, Nils Blüthgen, Hanspeter Herzel

**Affiliations:** 1 Institute for Theoretical Biology, Humboldt Universität zu Berlin and Charité Universitätsmedizin Berlin, Berlin, Germany; 2 Department of Systems Biology, Institute for Biomedical Genetics (IBMG), University of Stuttgart, Stuttgart, Germany; 3 Stuttgart Research Center for Systems Biology (SRCSB), University of Stuttgart, Stuttgart, Germany; 4 Biochemistry Center, Universität Heidelberg, Heidelberg, Germany; 5 Division of Theoretical Systems Biology, German Cancer Research Center (DKFZ), Universität Heidelberg, Heidelberg, Germany; 6 Laboratory of Chronobiology, Institute for Medical Immunology, Charité Universitätsmedizin Berlin, Berlin, Germany; 7 Institute of Pathology, Charité Universitätsmedizin Berlin, Berlin, Germany

**Keywords:** network switches, ultrasensitivity, bistability, feedback loops, circadian clock, mathematical models

## Abstract

Circadian rhythms are generated by complex interactions among genes and proteins. Self-sustained ∼24 h oscillations require negative feedback loops and sufficiently strong nonlinearities that are the product of molecular and network switches. Here, we review common mechanisms to obtain switch-like behavior, including cooperativity, antagonistic enzymes, multisite phosphorylation, positive feedback, and sequestration. We discuss how network switches play a crucial role as essential components in cellular circadian clocks, serving as integral parts of transcription-translation feedback loops that form the basis of circadian rhythm generation. The design principles of network switches and circadian clocks are illustrated by representative mathematical models that include bistable systems and negative feedback loops combined with Hill functions. This work underscores the importance of negative feedback loops and network switches as essential design principles for biological oscillations, emphasizing how an understanding of theoretical concepts can provide insights into the mechanisms generating biological rhythms.

## Molecular and network switches

Information processing in the physicochemical space and in biological systems depends on molecular entities that exist in two or more defined states. Molecular or conformational switches, involving changes in the 3-dimensional structure of molecules, such as proteins or lipids, lead to alterations in their physicochemical properties in a way that its function within a biological pathway is significantly altered. These molecular switches, often manifested as modifications within macromolecules at specific sites, result in conformational changes and serve as fundamental components of what we term ‘network switches’. Network switches, as we define here, are regulatory biochemical modules characterized by steep input-output relationships crucial for maintaining homeostasis, responding to external stimuli, and coordinating complex cellular activities, ultimately governing pathway activation and cell fate. This review primarily focuses on network switches and on their essential role in the generation of circadian rhythms. Illustrations of network switch-like input-output relations and the underlying mechanisms contributing to such dynamics are provided in Box 1.

Box 1 **Ultrasensitive or ‘switch-like’ stimulus-response relationships and their underlying mechanisms**

**Figure 1:**
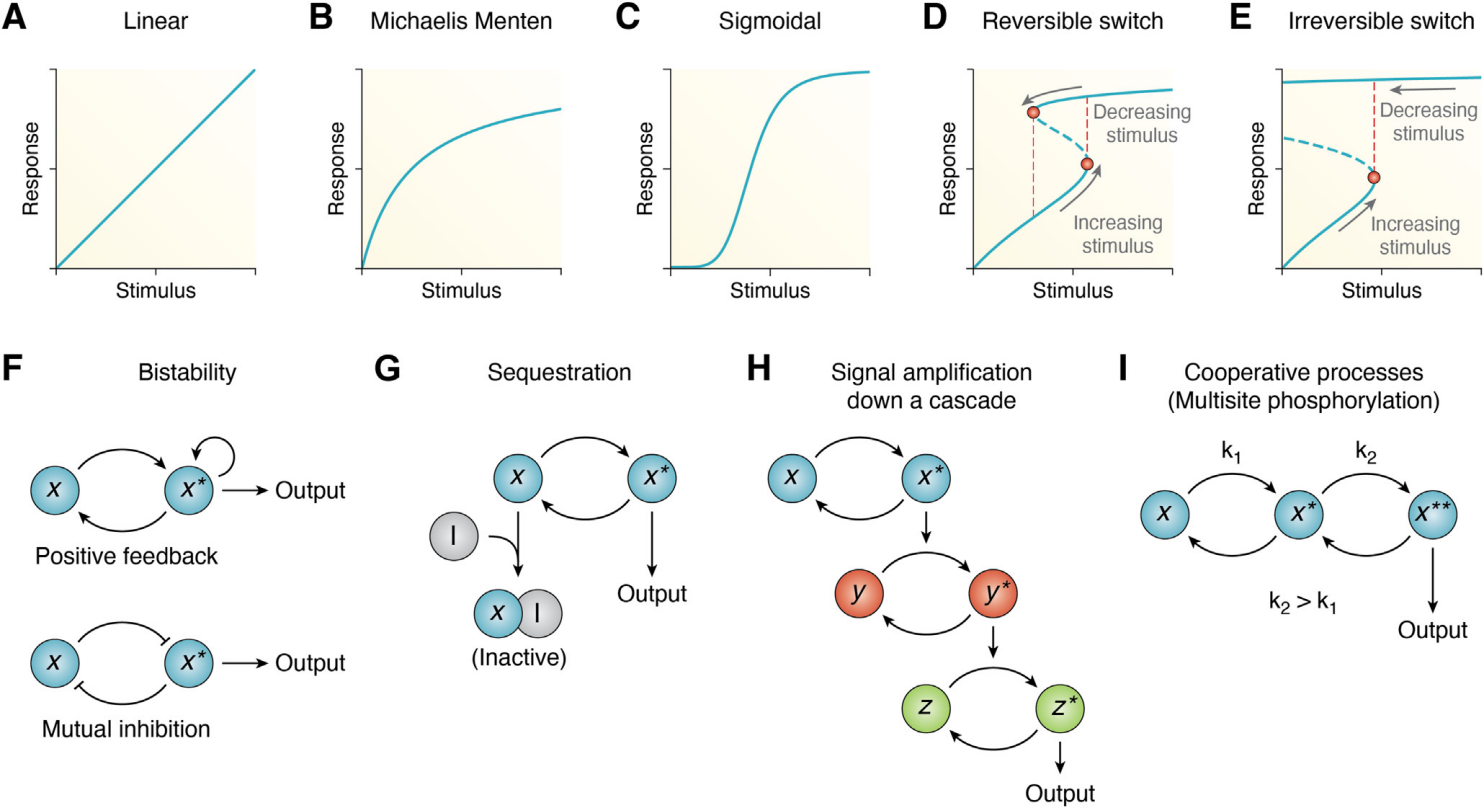
**Representative examples of different signal-response relationships and biochemical modules that generate network switches.***A*, linear signal-response curve, (*B*) hyperbolic Michaelis–Menten, (*C*) hill-type sigmoidal ultrasensitivity, (*D*) reversible bistable switch, and (*E*) irreversible bistable switch. Response curves represented by (*C*–*E*) serve as examples of network switches. (*D*) and (*E*) serve as examples of bistable systems, where two stable steady states coexist for certain values of stimulus. Depending on whether the stimulus is increased or decreased, the system will follow the lower or upper curve, respectively. Such loop-like curves indicate the presence of hysteresis. The unstable steady state separating both stable branches is shown with a *dashedblueline*. Bifurcation points in which the steady states change stability are shown in *red*. In the *bottom row*, different biological motifs that can give rise to switch-like dose-response curves are shown: (*F*) bistability through positive feedback or mutual inhibition, (*G*) sequestration, (*H*) signal amplification along a cascade, and (*I*) cooperative processes. In all panels, *x* and *x*^*∗*^ (or *y* and *y*^*∗*^; *z* and *z*^*∗*^) represent the inactive and active molecule counterparts, respectively, that are responsible for the output response.

In molecular and cellular biology, ultrasensitivity describes an output response that is more sensitive to stimulus change than a linear response or a hyperbolic Michaelis–Menten-like response (Fig. 1, *A* and *B*). The quantification of ultrasensitivity is often performed by fitting the sigmoidal Hill equation:

(1) $$response\;=\;\frac{\;{stimulus}^{n}}{{EC}_{50}^{n}\;+\;{stimulus}^{n}}$$

where the Hill exponent n reflects the steepness of the sigmoidal dose-response curve. If *n* = 1, the Hill function reflects simply the Michaelis–Menten-like stimulus-response relation.

Ultrasensitive curves correspond to monostable, yet steeply sigmoidal, steady state dose-response curves (Fig. 1*C*). This means that at every given stimulus value, only one specific response is the output. There are mechanisms, however, that can give rise to a discontinuous switching mechanism between two coexisting steady states. These systems are termed bistable, and bistability is widely believed to play a vital functional role in gene regulatory networks (56, 168, 169, 170, 171), cell differentiation (172, 173), cell cycle regulation (19, 174), lineage commitment during development (175), or exit from quiescence in mammalian cells (176). Moreover, a bistable switch can exist as a two-way reversible (toggle) switch (150) (Fig. 1*D*) or as a one-way irreversible switch (Fig. 1*E*). Toggle switches are reversible but exhibit hysteresis. This means that after the switch has been activated by a sufficient increase in stimulus—causing the system to reach the upper steady state—a significantly larger decrease in input stimulus is necessary to bring the system back to its lower steady state. On the other hand, one-way irreversible switches can never return the system to its lower steady state once the switch has been triggered. With ‘switch-like behavior’, we refer to both ultrasensitive and bistable systems (reversible or irreversible).

Quantitative studies of signal transduction systems have shown that ultrasensitive and bistable switch-like responses are commonplace in cell signaling. In Figure 1, *F*–*I*, we summarize some biological motifs that can contribute to such switching dynamics. In Box 2 and Box 3, we illustrate specific mechanisms by which some of these modules generate ultrasensitivity or bistability.

Box 2 **Modeling cooperative multisite phosphorylation and oxygen binding to hemoglobin leads to strong ultrasensitivity**

Many proteins have multiple phosphorylation sites and may require multiple phosphorylation events to be activated (or inactivated). This multistep process can lead to an ultrasensitive response, ultimately amplifying the response in cellular signaling pathways.

**Figure 2:**
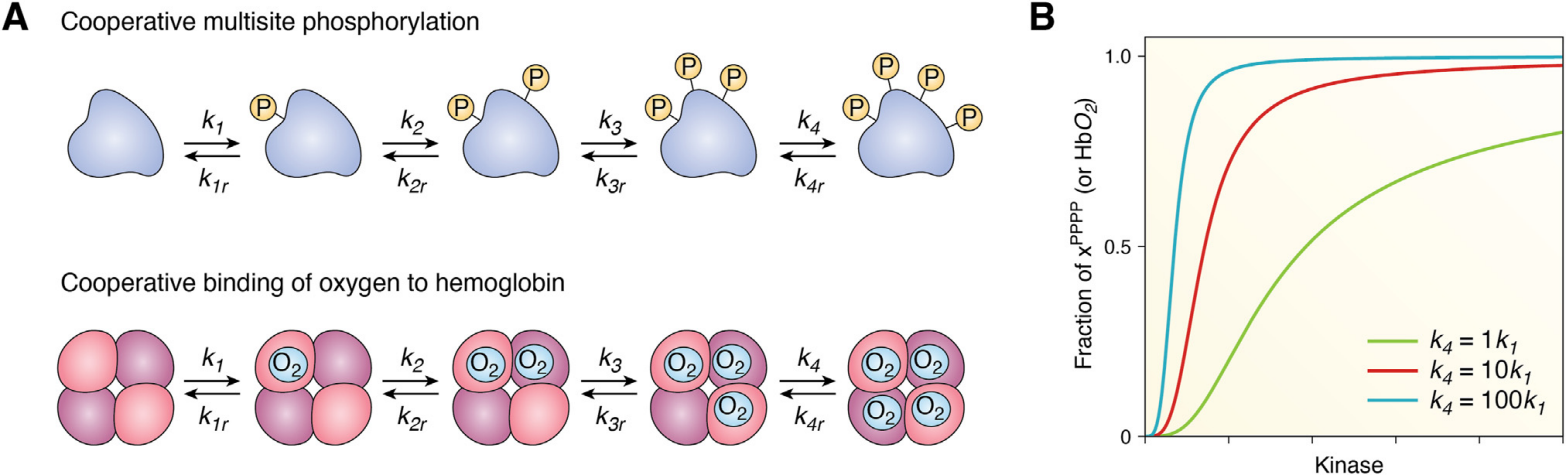
**Ultrasensitivity through cooperative multisite phosphorylation or cooperative binding of oxygen to hemoglobin.** Cooperative multisite phosphorylation (*A*, *top row*) or cooperative binding of oxygen to hemoglobin (*A*, *bottom row*) can lead to strong network ultrasensitive switches. The steady state response of the last element of the chain (the fully phosphorylated protein or hemoglobin with the four oxygen molecules bound) is shown as a function of the kinase or the O__2__ concentration (*B*). The green curve shows the response if all phosphorylation/binding reactions occur at the same rate, but the ultrasensitivity becomes greater if cooperativity in the phosphorylation/binding is assumed (*red curve*: *k*__*4*__ = 10*k*__*1*__, *blue curve*: *k*__*4*__ = 100*k*__*1*__), as seen by the increasing Hill exponents of Hill functions fitted to all three curves: n = 2.3 (*green*), n = 2.9 (*red*), and n = 3.4 (*blue*). All parameters in Equation 2 are set to 1 for simplicity (*i.e.*, phosphatase concentration, all reverse and forward reactions except *k*__*4*__, whose value changes depending on the degree of cooperativity assumed).

Let us suppose we have a protein that needs four sequential phosphorylation events to get activated (Fig. 2*A*), that is, there is a specific order of steps. Also, for simplicity, we suppose there is only one monophosphoform. Finally, we assume that the phosphorylation and dephosphorylation reactions can be described by mass action kinetics (*i.e.*, the kinases and phosphatases do not operate under saturating conditions). These series of reactions can be described by the following ODEs:

$$\frac{dx}{dt}\;=\;-\;k_{1}\;kinase\;x\;+\;k_{1r}\;ppase\;x^{P}$$

$$\frac{dx^{P}}{dt}\;=\;k_{1}\;kinase\;x\;\;-\;k_{1r}\;ppase\;x^{P}\;-\;k_{2}\;kinase{\;x}^{P}\;+k_{2r}\;ppase\;x^{PP}$$

(2) $$\frac{dx^{PP}}{dt}\;=\;k_{2}\;kinase\;x^{P}\;-\;k_{2r}\;ppase\;x^{PP}\;-\;k_{3}\;kinase{\;x}^{PP}\;+\;k_{3r}\;ppase\;x^{PPP}$$

$$\frac{dx^{PPP}}{dt}\;=\;k_{3}\;kinase\;x^{PP}\;-\;k_{3r}\;ppase\;x^{PPP}\;-\;k_{4}\;kinase{\;x}^{PPP}\;+\;k_{4r}\;ppase\;x^{PPPP}$$

$$\frac{dx^{PPPP}}{dt}\;=\;k_{4}\;kinase{\;x}^{PPP}\;-\;k_{4r}\;ppase\;x^{PPPP}$$

where *kinase* and *ppase* represent the concentrations of kinase and phosphatase; *k*_*1*_, *k*_*2*_, *k*_*3*_ and *k*_*4*_ represent the rate constants for the phosphorylation reactions and *k*_*1r*_, *k*_*2r*_, *k*_*3r*_, and *k*_*4r*_ represent the rate constants for the dephosphorylation reactions. This system of equations can be solved numerically in a way that we can investigate how the steady state concentration of the fraction of active fully phosphorylated protein (*x*^*PPPP*^) changes as a function of increasing kinase concentration. Moreover, we can explore how the curve changes when the last phosphorylation events are assumed to occur faster than the first ones, that is, if we assume that this is a cooperative process. The results show that ultrasensitivity becomes greater and the response becomes overall more switch-like and ultrasensitive (with higher effective Hill exponents *n*) if the last phosphorylation is assumed to be 10- or 100-fold more favorable than the first phosphorylation (Fig. 2*B*).

The same system of equations from Equation 2 can be used to describe the cooperative binding of multiple oxygen molecules to hemoglobin, where each step now describes the binding of an additional O_2_ molecule. In the scheme of Figure 2*A*, bottom row, the forward k parameters represent the binding of one O_2_ molecule to a hemoglobin subunit, and the reverse parameters represent the dissociation. Again, the binding of oxygen to hemoglobin follows an ultrasensitive steady state response curve only if cooperative binding is assumed, that is, if *k*_*4*_ > *k*_*1*_ (Fig. 2*B*).

Box 3 **Positive feedback loops lead to bistable switch-like responses**

A highly ultrasensitive response can be well approximated by a step function, which is one kind of switch—a monostable switch with just one stable steady state. However, systems with positive feedback loops can function as a different type of switch: as a bistable switch with hysteresis and, in some cases, irreversibility built into the system.

**Figure 3:**
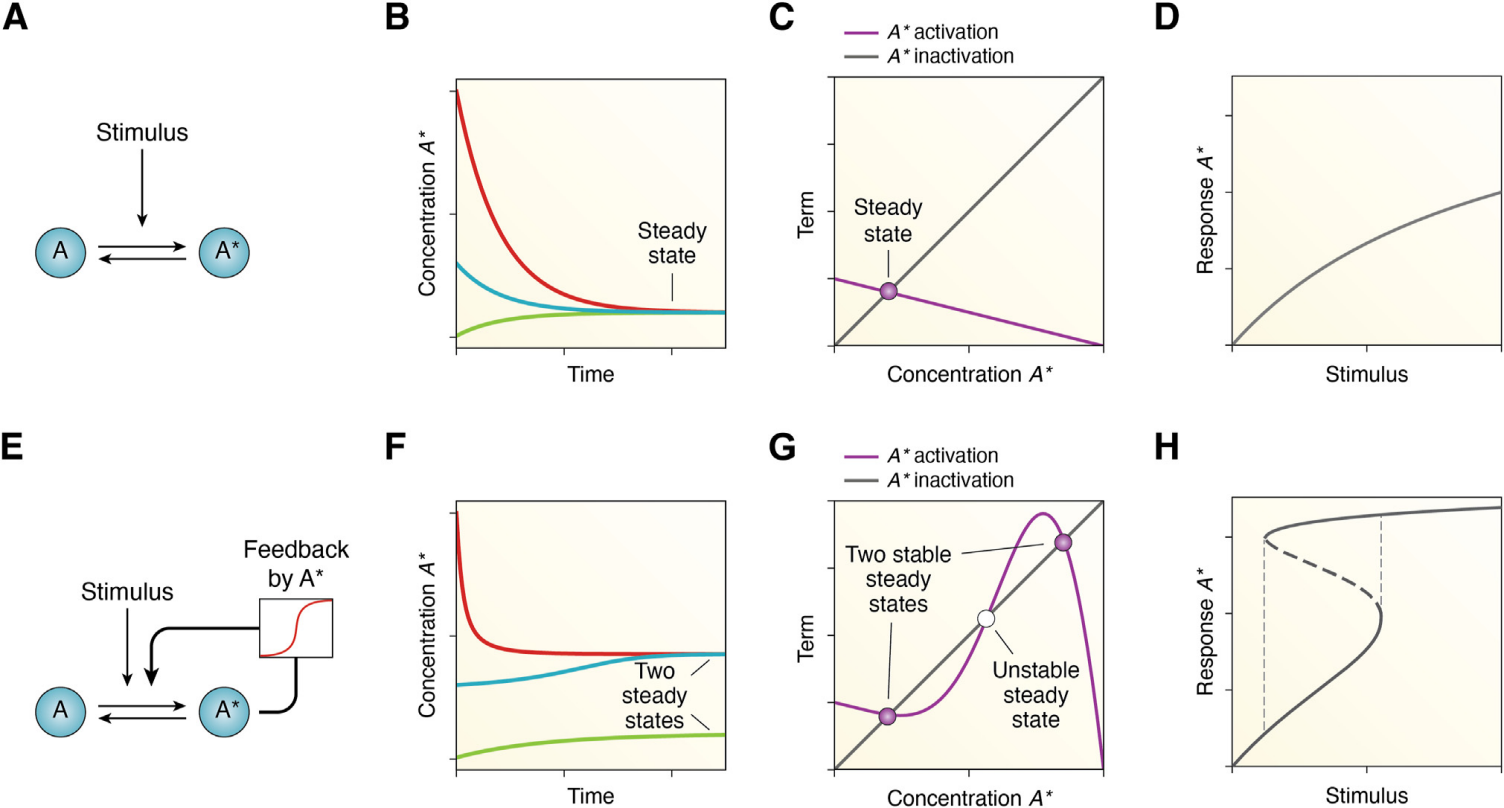
**Bistability depends on an ultrasensitive positive feedback.***Top row*, (*A*), scheme of a system without positive feedback where A is reversibly activated to A^∗^ by a stimulus. *B*, this system is monostable, and different starting initial conditions (shown in different colors) approach one steady state. *C*, the stable steady state of A^∗^ can also be found graphically, at the points where the line representing the activation term of A^∗^ as a function of A^∗^ (*purple*) intersects with the inactivation term of A^∗^ (*gray*). *D*, The stimulus-response curve is Michaelian. *Bottom row*, (*E*), same motif as in (*A*), now with a positive feedback where A^∗^ stimulates its own production. *F*, this system is now bistable, and different initial conditions (shown in different colors) converge in one or the other stable fixed point over time. *G*, the two stable steady states of A^∗^ (filled points) can be found graphically at the points where the activation term (*purple*) equals the inactivation term (*gray*) and are separated by an unstable steady state (unfilled point). *H*, the dose-response curve of this network becomes a reversible switch, represented by the hysteresis curve. Results are obtained for numerical integration of Equation 3 (*top row*) or Equation 4 (*bottom row*) for the following parameter values: *A*_*tot*_ = 1, *n* = 5, *d* = 0.01, *K* = 1, *f* = 0.15, and *stimulus* = 0.0025 (or changing stimulus values for panels *D* and *H*).

Here, we begin with a system with no positive feedback, in which a stimulus activates the conversion of a molecule *A* into its active state *A*^*∗*^ (Fig. 3*A*), described by the following ODE:

(3) $$\frac{dA^{*}}{dt}\;=\;stimulus\left(A_{tot}\;-\;A^{*}\right)\;-\;dA^{*}$$

where *d* represents the deactivation rate of *A*^*∗*^ back to *A*.

If we plot how, for a given stimulus, different starting conditions of *A*^*∗*^ change over time, they all approach the steady state (Fig. 3*B*), which is the value at which the activation term of *A*^*∗*^ equals the inactivation term of *A*^*∗*^ (Fig. 3*C*). By changing the values of the stimulus gradually and finding the steady state of *A*^*∗*^, we can determine the dose-response curve of this system: a hyperbolic Michaelian curve (Fig. 3*D*).

Let us now incorporate into the same system a positive feedback where the active product *A*^*∗*^ promotes its own production from *A*, and we will assume that there is ultrasensitivity in the feedback loop and that the feedback is proportional to a Hill function of *A*^*∗*^ (Fig. 3*E*). The ODE of this modified system then is:

(4) $$\frac{dA^{*}}{dt}\;=\;stimulus\left(A_{tot}\;-\;A^{*}\right)\;+\;f\frac{{\left(A^{*}\right)}^{n}}{K^{n}\;+\;{\left(A^{*}\right)}^{n}}\left(A_{tot}\;-\;A^{*}\right)\;-\;dA^{*}$$

In this system, there are two stable steady states and, for a given stimulus strength, whether one of them or the other is approached will depend on the initial conditions: some starting points are ‘attracted’ to the lower steady state, but others are attracted to the higher *A*^*∗*^ steady state (Fig. 3*F*). The stable steady states can be found graphically, in those points where the activation term equals the inactivation term of *A*^*∗*^ (Fig. 3*G*). If we now increase the stimulus gradually and find the steady state of the system for that particular value of stimulus, then a bistable switch can be found (Fig. 3*H*), and the stimulus-response curve splits into two curves: one representing the amount of stimulus needed to induce the system to its ‘on’ state, the other representing the amount of stimulus needed to maintain the system on its ‘on’ state. At this point, we say that the system is bistable for some values of stimulus, that is, there are two discrete stable steady states for some values of stimulus, and the system shows hysteresis. This means that the dose-response relationship is a loop rather than a curve.

If one eventually forces the feedback to be strong enough by increasing *f* from Equation 4, the switch can become irreversible and the system will stay at the ‘on’ state even if the stimulus is decreased to zero (see Fig. 1*E*).

Both molecular switches and network switches play fundamental roles in regulating biological processes. Molecular switches involve conformational changes at the individual molecule level, altering binding affinity, enzymatic activity, or interaction with other molecules, ultimately enabling toggling between different functional states. Examples include RAS-GDP/GTP transitions (1), prion switching (2), NFAT1 activation (3, 4), or rhodopsin stimulation (5). Network switches, on the other hand, refer to the resulting alteration in the biological pathway that such molecular switches induce. In other words, molecular switches can initiate major network switches, including pathway activation, checkpoint crossing, gene activation, and cell fate decisions—processes that are all characterized by switch-like input-output relationships.

## Mechanisms by which cells undergo ‘switching’

Stimulus-response curves of signal transduction cascades are often nonlinear and governed by switch-like relationships, where small alterations in the stimulus can elicit large changes in the response. This section reviews the importance of network switches in signal transduction, with a focus on the major mechanisms that generate switch-like input-output relationships.

### Cooperativity and multisite phosphorylation

The concept of nonlinear input-output relationships has been a topic of early discussion in the context of enzymatic reactions or binding of ligands to proteins. A famous textbook example is the binding of O_2_ to hemoglobin (6, 7, 8, 9), in which the affinity of the binding sites in hemoglobin for oxygen increases upon the binding of O_2_ to each subunit. Also the well-known Hodgkin-Huxley equations (10), describing how action potentials in neurons are initiated and propagated, incorporate strong cooperativity to reproduce experimental observations from the giant axon in squids. Another illustrative example is represented by proteins with multiple phosphorylation sites (11, 12), where priming of the kinase to the first phosphorylation site is slow but the kinase then functions cooperatively to phosphorylate the remaining sites (Box 2). Mechanistically, these nonlinearities in input-output relationships may emerge directly from coupled reactions following mass action kinetics (Box 2) or from the mass action kinetics of higher-order reactions. The ‘steepness’ of input-output relationships can be quantified with Hill exponents and those responses with a Hill exponent larger than 1 are commonly referred to as ultrasensitive (Fig. 1*C*) (8, 9, 12, 13, 14).

### Zero-order ultrasensitivity

In addition to multiple phosphorylation sites, single reversible enzymatic modifications can also give rise to a type of switch-like input-output relationship that has been termed ‘zero-order ultrasensitivity’ (15). Here, the antagonistic enzymes that act to modify a substrate (*e.g.*, a kinase and a phosphatase in a phosphorylation-dephosphorylation monocycle) do not follow mass action kinetics but are instead saturable and governed by the Michaelis–Menten equation. The fraction of substrate in the modified state can undergo a sharp switching from near-zero to near-unity at a critical value of the ratio of the enzyme concentrations. This mechanism requires fast enzymatic reactions and saturated working ranges of the two antagonistic enzymes (9, 16, 17, 18).

### Amplification down a cascade

Cell fate decisions often arise as the final outcomes of long, complex signaling cascades. Examples include mitogen-activated protein kinase (MAPK) pathways, extensively studied by the Ferrell (19, 20, 21) and Blüthgen (22, 23) groups, as well as the sequential cleavages of caspases during apoptosis (24, 25, 26). From a kinetic perspective, multisite phosphorylations can also be conceptualized as cascades of enzymatic reactions (12, 27). Ferrell and Ha review in (12) the impact of ultrasensitive responses in a three-step signaling cascade such as the MAPK cascade. Without ultrasensitivity in how the individual components respond to their upstream stimulus, that is, for Hill exponents *n* = 1 (Equation 1), a large input stimulus results in a weak final output response. Introducing ultrasensitivity with *n* ≥ 2 ensures that the magnitude of the input signal is preserved or even amplified in response to weak input signals, transforming them into decisive, switch-like outputs. These mathematical results underscore the importance of ultrasensitivity in preventing signal deterioration and facilitating signal amplification in signaling cascades.

### Positive feedback, bistable systems, and hysteresis

An additional generic mechanism that can contribute to switch-like behavior and amplify small input stimuli is positive feedback. There are manifold examples of positive feedback loops in the context of cellular networks: Cdc25, APC, and Wee1-mediated feedback in cell cycle checkpoints (19, 28, 29, 30, 31, 32, 33, 34, 35), calcium-induced calcium release (36, 37, 38), EGF receptor signaling (39, 40), p53 regulation (41, 42), or transcriptional feedback loops in cell differentiation processes regulated through MyoD (43, 44) and GATA-3 (45). Positive feedback loops often play a crucial role in inducing cell fate decisions characterized by bistability (29, 33, 46, 47) or irreversible switching (21, 28) (see also Box 1).

Positive feedback loops are evident in examples where downstream targets activate upstream inputs. Nevertheless, alternative mechanisms such as double negative feedback loops or inhibition of inhibitors (Fig. 1*F*) can also be regarded as positive feedback loops (26, 48, 49). Well-known examples of these motifs include the mutual inhibition of Th1 and Th2 cells (50, 51, 52) or the X-chromosome inactivation (53). Additionally, a decrease in degradation processes can also act as a hidden positive feedback mechanism (54, 55).

Positive feedback loops frequently give rise to bistable systems, a condition where multiple stable steady states coexist within a system and that can allow for sudden jumps between the stable states (Box 3). Notable examples of bistable systems resulting from positive feedback circuits include the lac operon (56), the phage lambda decision switch (57), cell fate determinations during oocyte maturation in *Xenopus* (19, 21, 29), and various cell differentiation processes. Transitions between the stable states can be induced by external perturbations, as demonstrated by the synthetic toggle switch engineered by Gardner (49) or by gradual increases of external parameters such as growth factors (58, 59) or antigen stimulations (52, 60) (see Box 1 and Box 3). Moreover, slowly varying parameters can lead to sudden transitions in bistable cell cycle circuits, thereby establishing checkpoints for cell cycle progression (19, 21, 29, 61). In some cases, these jumps are irreversible (*e.g.*, in cell differentiation); in other cases, backward transitions can be induced by reversing the parameter changes (Box 1). This phenomenon is known as hysteresis (Box 1 and Box 3), where forward and backward transitions occur at different parameter values, and it holds particular relevance in biochemical and physiological processes, including biological oscillations.

### Sequestration

In reaction networks, enzymes frequently participate in multiple reactions and interact with multiple binding partners. As a result, there can be instances of crosstalk within these networks that may not be apparent when only explicit positive and negative regulations are considered. The phenomenon of sequestration (see Box 1), where enzymes are effectively ‘sequestered’ or bound by specific molecules, can give rise to hidden positive or negative feedbacks (62), which in turn contribute to ultrasensitivity and bistability. Notable examples of such sequestration-driven switch-like responses are the cleavage of caspases in apoptosis (26), phosphorylation in the MAPK cascade (58), phosphorylation of KaiC tetramers in the circadian clock of cyanobacteria (63), and the displacement of the activators CLOCK:BMAL1 by the repressors PER2:CRY1 in the mammalian circadian clockwork (64, 65, 66, 67, 68). In the following sections, we discuss how network switches contribute to rhythm generation within circadian clocks.

## Design principles of cellular biological rhythms

Biochemical oscillators are characterized by common design principles: delayed negative feedbacks and sufficient nonlinearities in the reaction kinetics (69) are needed to generate self-sustained oscillations. The required nonlinearities often arise from network switches. In this section, we link the features of transcription-translation feedback loops (TTFLs) with mechanisms of switch-like behavior, as discussed earlier.

### Oscillations require delayed negative feedback loops

Homeostatic regulations typically lead to gradual and monotonic growth or decay towards steady states which are governed by production and degradation balances. Rhythmic behavior requires, in contrast, periodic recurrences. This implies that negative feedbacks should exist in order to bring the concentrations of the clock elements back to their starting values. Moreover, the negative feedback must be sufficiently delayed in time so that the biochemical reactions do not settle on a steady state (69). In the context of biological clocks, TTFLs allow this, as exemplified by circadian clocks (70, 71), by the somitic segmentation clock (72, 73), p53 pulses (74, 75), or NFκB oscillations (76, 77).

Time delays in the negative feedback can be created by long chains of intermediates between the ‘cause’ and the ‘effect’ of the negative feedback loop, but they can also arise through dynamical hysteresis or by introducing explicit physical constraints (69). In delay differential equations (DDEs), a type of differential equations in which the derivative of the unknown function at a certain time is given in terms of the values of the function at previous times (78), time delays are included explicitly. This type of mathematical modeling provides direct relationships between the delays and the oscillation period (79, 80, 81). Specifically, DDE and ordinary differential equation (ODE) models establish that the duration of the delay is often around one-quarter of the overall period (Box 4). This relationship indeed helps explain the characteristic frequencies observed in various systems, including periods of a few hours observed in human somitogenesis (72, 73, 82), p53 signaling (42, 75), and NFκB oscillations (76, 77).

Box 4 **Long delays and network switches are required for oscillations**

**Figure 4:**
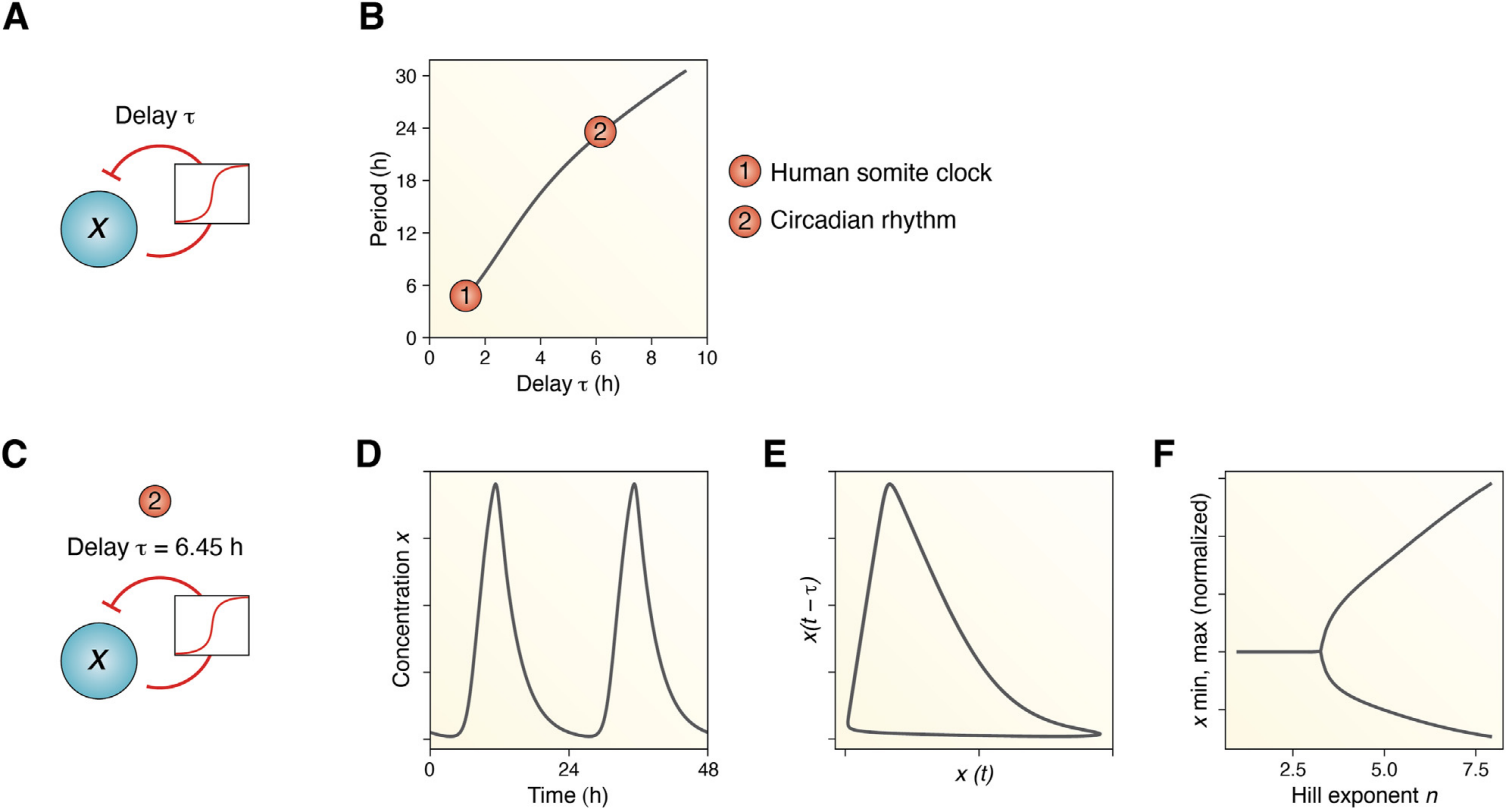
**Long delays of about a quarter of the oscillation period together with network switches are required to achieve self-sustained limit cycle oscillations.***A*, scheme of a simple delay-oscillator model, where a clock gene *x* represses its own transcription after a delay *τ*. The repression is modeled with a Hill-like term as described in Equation 5. *B*, relationship between the delay *τ* and the period of *x*. Delays of ∼1.6 h result in oscillations of 5 to 6 h period (the human segmentation clock could be an example of such oscillations), whereas circadian ∼24 h periods are obtained with longer delays of 6 to 7 h. Results are obtained through numerical integration of Equation 5 for *β* = 4, *n* = 5, *d* = 0.1, *K* = 1 and changing delays *τ*. *C*, scheme of the simple DDE model with *τ* = 6.45 h reproduces circadian oscillations. *D*, time series of the solution for *τ* = 6.45 h and (*E*) phase space, where *x* at the time *t − τ* is plotted *versus x(t)*. *F*, Hopf bifurcation diagram for changing Hill exponent *n*: it is observed how a strong nonlinearity of *n* > 3 is needed to generate limit cycle oscillations. Otherwise, oscillations dampen out and converge to a stable steady state. Results are obtained through numerical integration of Equation 5 for *β* = 4, *n* = 6.5, *d* = 0.1, and *K* = 1. DDE, delay differential equation.

Theoretical studies have shown that a single delayed negative feedback loop, combined with sufficient nonlinearity, can result in oscillatory behavior (78, 139, 177, 178) (see Fig. 4*A* for a scheme of the corresponding network motif). To capture the intermediate regulatory processes occurring within the delayed feedback loop, we can model such single negative feedback loop using a one-variable DDE with five parameters, including the delay *τ*, that accounts for the translation processes, post-transcriptional or post-translational modifications, nuclear import, etc., that biologically contribute to the delay:

(5) $$\frac{dx}{dt}\;=\;\frac{\beta }{K\;+\;x{\left(t\;-\;\tau \right)}^{n}}\;-\;dx$$

Results obtained through numerical integration of Equation 5 show that delays of approximately a quarter of the oscillation period are needed in order to generate stable limit cycle rhythms. Specifically, the human somite clock, that has a period of about 5 to 6 h (179), can be replicated with the DDE model if the delay is *τ* ∼ 1.6 h. Circadian oscillations, on the other hand, can be obtained with longer delays of *τ* ∼ 6 h (Fig. 4, *B* and *C*). The results for *τ* = 6.45 h are shown in time series (Fig. 4*D*) or in phase space (Fig. 4*E*), where delayed x(t), that is, *x(t−τ)*, is plotted against *x(t)* and the closed curve characteristic of limit cycles is illustrated.

This simple DDE model highlights the second essential feature that a system needs to generate self-sustained oscillations: the presence of sufficiently strong nonlinearities, with Hill exponents of at least 3 (Fig. 4*F*). Figure 4*F* shows the Hopf bifurcation diagram (180), where the peaks and troughs of our oscillating transcript *x* are shown as a function of increasing Hill exponent. For Hill exponents < 3, the system does not oscillate and instead converges to a steady state; thus, peaks and troughs of *x* are indistinguishable and a single point is plotted. On the other hand, Hill exponents > 3, indicative of steeper Hill switch-like curves, result in self-sustained oscillations of *x*, and therefore two points are plotted representing the peaks (upper branch) or troughs (lower branch) of *x*.

In contrast to these fast biological oscillations, the circadian clock operates on a longer timescale, with a periodicity of approximately 24 h. This implies that the required delay to generate stable oscillations must be longer, of at least 6 h (Box 4). Such a long delay cannot be easily attributed to transcription and translation alone, which for most genes happen in the order of minutes-hours (83, 84, 85). Consequently, specific mechanisms must have evolved to establish these lengthy and well-controlled delays. In *Drosophila*, the nuclear import of the clock proteins PER and TIM is suggested to be slow and delayed by about 5.5 h (86, 87). In *Neurospora* and mammals, phosphorylation cascades of intrinsically disordered proteins (IDPs) such as FRQ and PER2 have been suggested to contribute to the long delays (88, 89, 90, 91, 92).

### Network switches allow self-sustained oscillations

We have seen in the previous section how negative feedback loops are required for oscillatory dynamics. Nevertheless, they are not sufficient for the generation of stable self-sustained rhythms: strong nonlinearities are also needed (69). In the absence of nonlinear switch-like terms, linear systems exhibit dampened oscillations that do not persist over time. Circadian clocks are examples of self-sustained rhythms that do not dampen in many cases and, even without external zeitgebers (light-dark cycles, social cues, temperature cycles…), many organisms display persistent oscillations defined by an endogenous period and amplitude. Leise *et al.* (93) monitored for example single cell oscillations in fibroblasts and found that they were sustained for over more than 40 days with cell-specific periods and amplitudes.

These self-sustained oscillations are also termed stable limit cycles, and they are characterized by the fact that different initial conditions approach asymptotically to a closed curve in the phase space of relevant variables (94, 95). Thus, limit cycle oscillations require delayed negative feedbacks and sufficiently strong switch-like nonlinearities (Box 4). In the following section, we discuss some biochemical mechanisms that can contribute to long delays and nonlinear switch-like regulations in circadian TTFLs.

## Sources of network switches in the mammalian circadian clockwork

Circadian rhythms are ∼24 h oscillations which have evolved in most light-sensitive organisms as an adaptation to the daily cycle of light and dark. These clocks, which oscillate autonomously in the absence of external cues, allow organisms to anticipate changes in the environment and to coordinate their physiology, metabolism, and behavior around daily changing cues. Although circadian clocks seem to have evolved independently across the phylogenetic tree, fungi, plants, insects, and mammals share a common design principle: a TTFL. In this regulatory circuit, firstly, positive regulators stimulate the transcription of clock genes. These genes are then translated into proteins, and finally, some of these proteins act as negative regulators, suppressing the activity of the positive regulators (Box 5).

Box 5 **Design principles of circadian clocks in eukaryotic organisms, with focus on mammals: TTFLs**

**Figure 5:**
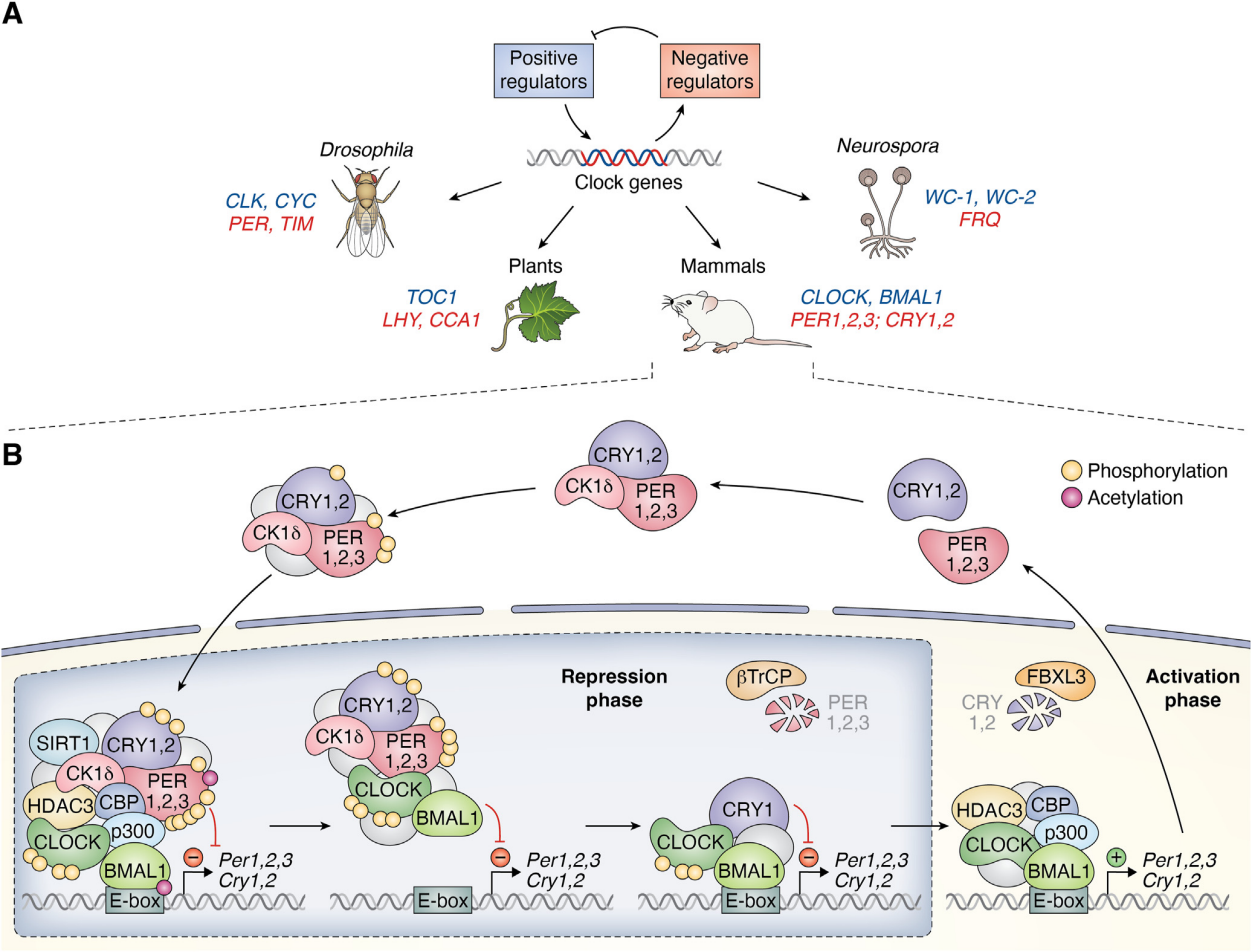
**Circadian clocks in eukaryotic organisms are controlled through a common mechanism: a transcription-translation feedback loop.***A*, TTFL circuitries in different kingdoms of life: positive regulators (*blue*) induce the transcription of clock genes which, when translated, produce negative regulators (*red*) that inhibit the positive arm of the loop therefore creating a negative feedback loop. *B*, scheme of the transcription-translation feedback loop in the mammalian circadian clockwork network. Core clock proteins and epigenetic regulators that have been identified as part of the macromolecular complexes through high-resolution biochemical experiments are depicted. Possible sources of switching are shown (phosphorylation cascades, sequestration partners, epigenetic modifiers) and described in the text.

Essentially all light-sensitive species have evolved circadian clocks as a response to the regular day-to-night changes in the environment. Circadian clocks are cell-autonomous oscillators, and although they have evolved independently in different eukaryotic species, they share a common regulatory architecture: TTFLs comprising three important ‘building blocks’. (i) Firstly, positive regulators promote the transcription of various clock genes, which then (ii) are translated into proteins, some of which, lastly, (iii) act as negative regulators and repress the activity of the positive regulators. The positive and negative regulators of the core clock differ across species. For example, in mammals, CLOCK and BMAL1 act as activators, and PER and CRY proteins are negative regulators; in fungi, the positive regulators are termed WC-1 and WC-2 and they promote the expression of the negative regulator FRQ (Fig. 5*A*).

We have reviewed in the main text some of the biochemical mechanisms that contribute to nonlinear and switch-like behavior that circadian clocks need to maintain their timekeeping properties. The cooperative interactions and mass action kinetics of high-order reactions among various clock proteins and transcription factors (PERs, CRYs, casein kinases, CLOCK, BMAL1…) within macromolecular complexes play a crucial role in generating the network switches that are needed for limit cycle oscillations. Moreover, phosphorylation, ubiquitination, and acetylation cascades involving CLOCK, PERs, or CRYs can generate highly steep responses (see Box 2), establishing a switch-like relationship between input and output that ensures accurate timing and robustness. Epigenetic regulation involving histone and protein acetylation (CBP, p300) and deacetylation (HDAC3, SIRT1) contribute to further control of clock gene transcription, potentially through sequestration processes, and thus also function as network switches. Figure 5 illustrates a schematic representation of the main players and modifications within the CLOCK:BMAL1–PER:CRY feedback loop. The dual model for E-box inhibition (65, 66) is shown: at the early repressive phase, the macromolecular complex including PER1-3, CRY1, CRY2, and CK1δ removes the CLOCK:BMAL1 activator complex from the E-box in a CRY-dependent manner (displacement type of repression). Later, CRY1 alone binds to the activation complex and directly represses the CLOCK:BMAL1 complex (blocking type of repression).

There are some examples of circadian clocks that do not fall into this ‘canonical’ mechanism of generating rhythmicity based on TTFLs, for example, some bacterial clocks, that will be discussed in a further section.

In mammals, the activators include CLOCK and BMAL1, while PER and CRY proteins function as negative regulators (see Box 5 and Fig. 5*A*). While this negative feedback loop plays a crucial role in the generation of self-sustained oscillations, it is not sufficient on its own. Network switches are also needed: oscillating systems require sharp threshold response curves to ensure that the oscillations do not dampen out and settle into a steady state. In the following, we will discuss how mechanisms of switch-like behavior such as cooperativity, phosphorylation cascades, and antagonistic enzymes contribute to oscillations in circadian TTFLs.

### Transcriptional cooperativity and sequestration

Transcriptional regulation of clock genes involves a highly dynamic and cooperative process. For instance, in mammals, the presence of E-boxes enhances transcription of clock genes when the transcription factors CLOCK and BMAL1 bind (Box 5). The DNA-bound complex recruits histone acetyltransferases and deacetylases (96, 97), which further regulate cooperatively the transcription of genes like *PER1*, *PER2*, *PER3*, and *CRY1*, *CRY2*. Several high amplitude rhythmic genes have even been shown to have tandem E-boxes in their promoters (98), suggesting even stronger cooperativity in their regulation. Additionally, there are other basic helix-loop-helix transcription factors capable of binding to E-boxes. For example, DEC1 and DEC2 can act as stoichiometric inhibitors (99), which can lead to sequestration-induced ultrasensitivity (12, 64). Moreover, single cell data have revealed that the transcriptional activity of E-box–containing promoters can be characterized as stochastic ‘on-off’ switches, giving rise to bursts in transcription (100, 101, 102).

Genome-wide ChIP-Seq studies mapping the temporal patterns of DNA binding by the core circadian factors have provided evidence for the existence of two distinct repressive complexes on E-boxes. During the early phase of repression, PER1, PER2, PER3, CRY1, CRY2, and the dedicated clock kinase CK1δ all interact with CLOCK:BMAL1 on DNA; and in a later phase, CRY1 alone is found to be bound to CLOCK:BMAL1 on DNA (103) (see Box 5 and Fig. 5*B*). Large-scale purification of native clock protein complexes from mouse liver throughout the day has expanded our understanding of the size and complexity of these macromolecular complexes, along with how their composition changes throughout the circadian cycle (66, 104). The current paradigm is that repression of CLOCK:BMAL1-activated transcription occurs by two mechanisms: a macromolecular complex including PERs and CK1δ which, in a CRY-dependent manner, removes the CLOCK:BMAL1 activator complex from the E-box (termed “displacement-type repression” in the literature) or CRY1 alone binding to the activation complex and directly repressing it (“blocking-type repression”) (65, 66, 67, 68, 105, 106, 107, 108). The displacement type of inhibition might act as a source of sequestration because the repressor complex, containing PER and CRY proteins, holds the activator complex and prevents it from binding to the E-boxes.

### Protein phosphorylation

Within mammalian TTFLs, additional levels of nonlinear dynamic regulations occur at the protein level that can contribute to switch-like behavior. PER and CRY proteins have been shown to form huge cytoplasmic and nuclear complexes of up to 1.9 MDa (104). Nevertheless, it is unclear whether all of the interaction partners of CRY and PER present in the large MDa complexes are integral clock components, partners involved in non-canonical clock or non-clock functions or simply as ‘fellow travellers’, as the partners are not reproducible using different methods (66). Additionally, the stability of clock proteins and their nuclear translocation is regulated by multiple phosphorylations (92, 109, 110). PER2, in particular, is an IDP with numerous phosphorylation sites and many interaction partners, including casein kinases (CK1δ and, CK2) and ubiquitin E3 ligases (71, 111, 112) (see Box 5). The slow and gradual phosphorylation of IDPs is a likely mechanism to provide the required long delays in circadian TTFLs (90, 91, 113).

Specific phosphorylations of PER2 constitute network switches that allow its import to the nucleus: only upon priming of kinases and phosphorylation of certain amino acid residues can PER2 be translocated to the nucleus (110). Phosphorylation of PER2 by CK1 is integral to the regulation of PER2 stability and circadian period (108, 114, 115). In the familial sleep phase syndrome, mutations in a phosphorylation site of PER2 (89) or in the kinase of PER2 (116), CK1δ, destabilize PER2 and shorten the circadian period. However, the picture is not quite that simple: CK1δ and CK1 both bind to PER2 and can either stabilize or promote degradation of PER2, depending on the exact modification site. Phosphorylation of the ‘phosphodegron’ site (109) promotes recruitment of the E3 ligase β-TrCP and thus degradation, whereas phosphorylation of the familial sleep phase syndrome region (89) delays degradation. This interplay constitutes the ‘phosphoswitch’ model (92, 109, 117, 118), which proposes that the balance of phosphorylation between the stabilizing and degrading regions is what determines overall PER2 half life.

### Epigenetic regulation

The mammalian circadian clock operates through interlocked feedback loops. In the primary loop, PER and CRY proteins inhibit the activation of their own transcription induced by CLOCK:BMAL1. Macromolecular assemblies containing activator and repressor complexes interact with various epigenetic regulators (96, 103, 119, 120, 121). In an additional loop, *BMAL1* expression is antagonistically regulated by the core clock proteins ROR and REV-ERBs (122), which are in turn also regulated epigenetically through large complexes (121, 123). The epigenetic landscape of the core clock is rather complex and there are many regulators activating or repressing the activity of core clock proteins; some of them even have opposite effects (activation/repression) depending on the phase of the circadian cycle and the binding partners that are present.

The activator complex with CLOCK:BMAL1 includes the histone acetyltransferases CBP and p300. These proteins rhythmically acetylate H3 histones (96), and H3 acetylation has in turn been shown to be a potential target of the inhibitory action of CRY proteins (96). Additionally, HDAC3 is a histone deacetylase that plays an important role in both activation and repression phases of the expression of E-box–driven genes. During the activation phase, HDAC3 promotes BMAL1 stability, but during the repressive phase, HDAC3 prevents CRY1 degradation and facilitates the association of BMAL1 and CRY1, thus strengthening CRY1-mediated repression (124) and ultimately contributing to robust rhythms in the expression of E-box–driven genes. HDAC3 plays an additional role in stabilizing the secondary loop: it functions as a co-repressor for REV-ERBα and helps in the inhibition of the expression of BMAL1. SIRT1, a histone deacetylase, interacts with CLOCK:BMAL1 in a circadian manner and promotes the deacetylation and degradation of PER2 (119). Furthermore, CLOCK has been identified as a histone acetyltransferase and acetylates its partner BMAL1 (125). The dynamic interplay between these opposing histone modifiers has been extensively reviewed in (97, 120, 121, 126) and likely serves as sequestration-based modules that contribute to the ‘on-off’ switch mechanism for clock gene transcription.

In summary, switch-like behavior in mammalian TTFLs arises from the integration of multiple mechanisms, including cooperativity of transcription factors that turn on and off transcription rhythmically throughout the circadian cycle (65, 100, 103, 122), phosphorylation cascades, dynamic complex formations, sequestration processes (66, 99, 106), and antagonistic epigenetic regulation (96, 97, 103, 119, 120, 121). Although a comprehensive quantitative understanding of these switches is currently lacking (65, 112), mathematical modeling offers a valuable approach to explore the necessary and sufficient conditions by which cells can generate stable limit cycle oscillations to keep circadian time (93, 127, 128, 129, 130).

## Network switches in models of the circadian clock

Mathematical models of biological rhythms have been applied to manifold systems: predator-prey population models (131, 132, 133), glycolytic oscillations (134, 135, 136), or circadian clocks (64, 137, 138). One of the early models by Goodwin (139) has been widely used in circadian clock research and focuses on delayed negative feedback loops in gene expression, where the final product of a 3-step chain of reactions *x → y → z* inhibits the production of the first component. (Note how this representation is a more ‘mechanistic’ implementation of the one-variable delay model presented in Box 4, where the delay *τ* is substituted by the chain of intermediates *y → z*). In the Goodwin model, relatively small degradation rates are needed to achieve the necessary long delay required for self-sustained oscillations. This model contains only one nonlinearity, namely the inhibition of transcription of *x via* the inhibitor *z*, which must be a strong inhibitory ultrasensitive switch (with a Hill exponent of at least 8) for the model to display self-sustained oscillations (Box 6).

Box 6 **Goodwin model for circadian oscillations and switch-like inhibition of clock gene expression**

**Figure 6:**
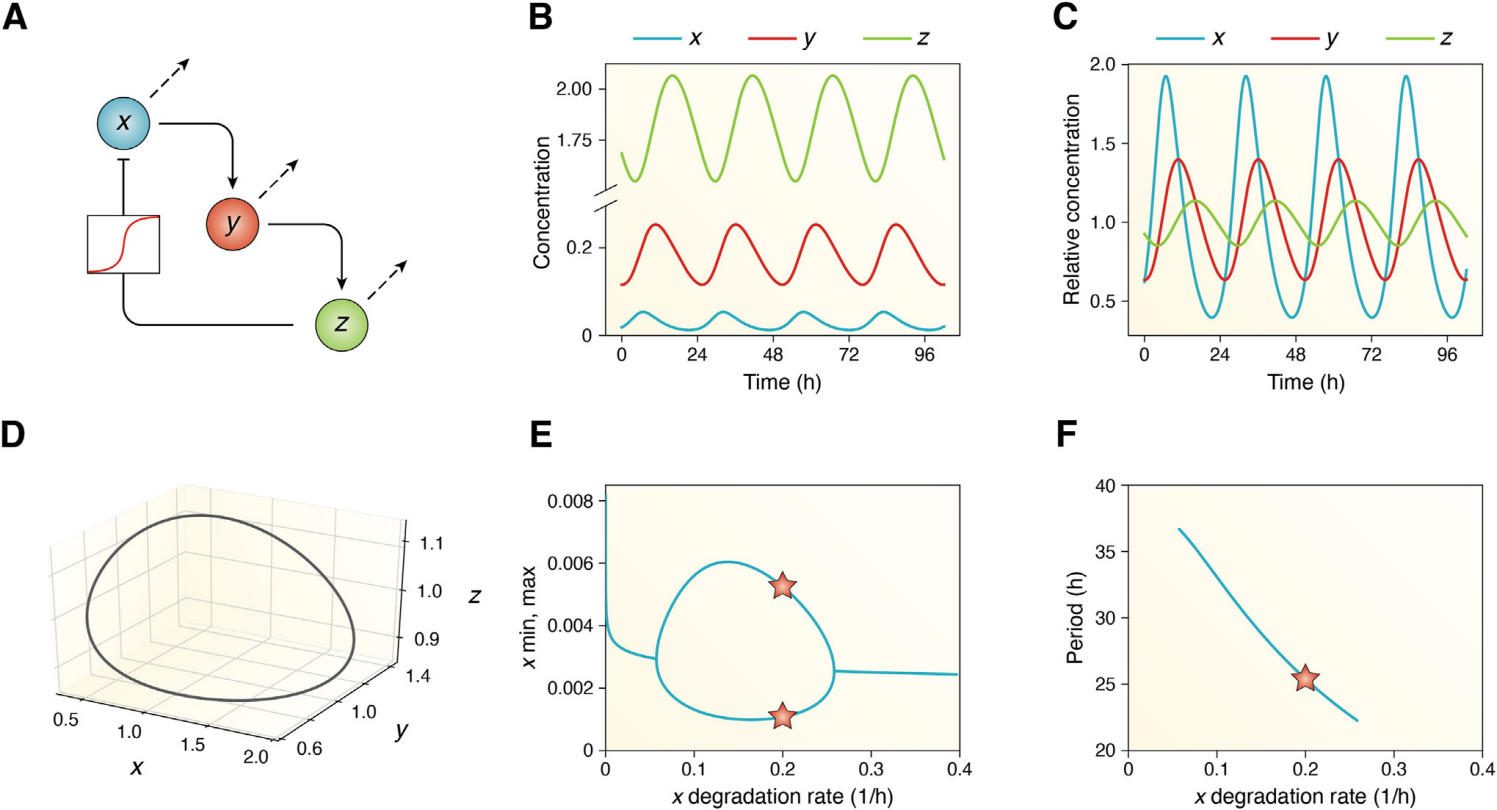
**Goodwin model for circadian limit cycle oscillations.***A*, scheme of the 3-variable model; note the sigmoidal curve with which *z* is modeled to repress *x*. *B* and *C*, time series, in absolute concentration terms (*B*), or after normalizing each rhythmic variable to its mean (*C*). *D*, phase space of the normalized *x*, *y*, and *z* variables of the Goodwin model. *E*, Bifurcation diagram as a function of increasing the degradation rate of *x*. *F*, within the range of *x* degradation rate that generates self-sustained oscillations, the period decreases monotonically. In (*E*) and (*F*), *stars* indicate the default parameter values in Equation 6. Results are obtained for numerical integration of Equations 6 for the following parameter values: *k*_*1*_ = *k*_*3*_ = *k*_*5*_ = 1, *K* = 1, *k*_*2*_ = 0.2, *k*_*4*_ = 0.15, *k*_*6*_ = 0.1, and *n* = 9.5.

One of the simplest and most widely used oscillator models is the one introduced by Goodwin (139). Developed in 1965, Goodwin’s model predates the comprehensive understanding of molecular mechanisms underlying circadian clocks. This model is based on delayed negative feedback loop, where the end product of a sequential chain of three reactions inhibits the production of the initial component (Fig. 6*A*). In the context of circadian clocks, this model can be interpreted as a clock gene mRNA *x* that gets translated into a clock protein *y* that then activates the repressor *z* which ultimately inhibits the transcription of *x* (note the similarity to the generic TTFL scheme from Box 5). The equations describing the oscillatory dynamics of the model are:

$$\frac{dx}{dt}\;=\;k_{1}\frac{K^{n}}{K^{n}+z^{n}}\;-\;k_{2}x$$

(6) $$\frac{dx}{dt}\;=\;k_{3}x\;-\;k_{4}y$$

$$\frac{dx}{dt}\;=\;k_{5}y\;-\;k_{6}z$$

where *k*_*1*_*, k*_*3*_*, k*_5_, represent the production rates of *x, y, z*, respectively; *k*_*2*_, *k*_*4*_, *k*_*6*_ represent the degradation rates; and where the repression that *z* exerts on *x* is modeled with a Hill function with Hill exponent *n*.

Numerical integration of the Goodwin model equations over time, with an appropriate parameter choice, can result in self-sustained limit cycle oscillations. The results can be plotted as a time series of the absolute concentrations of the variables (Fig. 6*B*), where it is clearly observed that the mean oscillating levels of *z* are higher than those of *y*, which are in turn larger than those from *x*. Note that when *z* is at its minimum, inhibition is negligible and *x* is at its maximum oscillating levels, whereas when *z* is at its peak, the transcription of *x* is close to its minimum. By normalizing these time series to their means, we can obtain the relative concentration of the three Goodwin species (Fig. 6*C*), and from this plot, it becomes evident that the relative amplitude of *x* is largest and that there is a phase delay between *x* and *z* of approximately 8 h. The solution can also be plotted in phase space, which is the illustration of the space of all possible states. Here, the characteristic closed curve of limit cycles appears (Fig. 6*D*).

Griffith demonstrated that the Hill exponent of the Goodwin model had to be sufficiently large (*n* = 8) for the model to generate self-sustained oscillators (181). Translated into biological words, this means that the *z*-mediated repression of *x* must be a very strong switch-like event—transcriptional cooperativity or multiple post-translational modifications might contribute to such an ultrasensitive step curve. But not only must the Hill exponent be > 8 to achieve stable oscillations, also the degradation rates of the three components must be tightly regulated and in a similar range of values. If any of the degradation parameters becomes too small or too large, the three-component feedback loop does not generate sufficient delay for oscillations. For our choice of parameters, the Goodwin model shows oscillations for values of *x* degradation rate between 0.05 and 0.25 (Fig. 6*E*): outside this range, the minimum and maximum of *x* overlap, indicating that the system stabilizes into a steady state instead of oscillating. Moreover, for this range of degradation values, the period decreases monotonically (Fig. 6*F*).

The Goodwin model has been extensively studied and fine tuned by Gonze and Abou-Jaoudé (18) and others to reduce the need of such high Hill exponent (*n* > 8), which has been argued not to be biologically meaningful. Subsequent modifications to the Goodwin model have proposed alternative mechanisms that may contribute to strong switch-like behavior. Longer reaction chains representing, for instance, multiple phosphorylations and complex formations allow for smaller Hill exponents (18, 62, 78, 140). Moreover, synergies with other feedback loops lead to biochemically more plausible models (54, 137, 138, 141). Extended versions of the Goodwin oscillator have been used to investigate temperature compensation in *Neurospora* (142), coupled SCN neurons (143), and the role of amplitudes in jet-lag recovery (144).

In an elegant study by Gonze and Abou-Jaoudé (18), the ultrasensitive inhibition switch modeled with a Hill function was replaced by ultrasensitive motifs achieved *via* multisite phosphorylation, zero-order ultrasensitivity, or by a double phosphorylation cycle giving rise to bistability. Such modules with multiple phosphorylation cycles have been studied by more scientists in the field (109, 110, 113, 145, 146). An alternative formulation of simple Goodwin-like systems is proposed by Kim and Forger (64). Here, the authors use a sequestration-based module to describe the negative feedback loop, in which the repressor sequesters the activator. They show that sharp thresholds are only achieved around a 1:1 stoichiometric balance between repressor and activator. In fact, in the mammalian clockwork, inhibition of transcriptional activity is achieved (at least partly) through the formation of a 1:1 stoichiometric complex between activators (CLOCK:BMAL1) and inhibitors (PERs and CRYs) in the nuclei of cells (104, 147). Taken together, these modeling results show that explicit Hill functions are strictly speaking not necessary to generate self-sustained oscillations, and one can rely on other strategies to achieve sharp switch-like responses.

Network switches play an additional role by not only contributing to the generation of oscillations but also effectively filtering the inherent spatiotemporal noise encountered by cells. In a recent study from Chae *et al.* (110), the authors show how a bistable phosphoswitch module facilitates precise nuclear entry of PER proteins and sharp transcriptional repression, despite the wide and variable time window of PER arrival at the perinucleus. This mechanism ensures robust and properly-timed circadian rhythms, even when cells vary in size or in cytoplasmic congestion. This way, network switches offer a means to filter cellular noise and enhance synchronization, contributing to coherent cellular physiology and to the generation of robust circadian rhythms.

In summary, endogenously generated self-sustained oscillations (*i.e.*, limit cycles) are ubiquitous in biological processes, ranging from glycolysis (135) and the frog egg cell cycle (148) to somitogenesis (72, 73) and circadian clocks (71, 112), with periods spanning from minutes to 1 day. In all these cases, negative delayed feedback loops and network switches are involved. This design is particularly clear in the Goodwin system (Box 6), a model that exploits a Hill-type ultrasensitive inhibition switch. However, additional mechanisms besides the Hill equation can still generate sharp switches: multisite phosphorylations, zero-order ultrasensitivity, sequestration, or bistability through a double phosphorylation cycle (18, 64, 149). These modules also contribute to synchronized and robust rhythms by filtering out the inherent noise that cells encounter in their environments (110). More examples of oscillator models with network switches can be found in comprehensive reviews and book chapters (9, 12, 13, 150, 151).

## Summary and discussion

Network switches in biological systems frequently display sigmoidal integral (∫)- or bistable S-shaped stimulus-response curves. These curves can be quantified using Hill exponents (*n*) to measure their steepness. By convention, Hill functions with *n* > 1 are termed ultrasensitive. This review illustrates different examples where biological systems exhibit these switch-like response curves, ranging from the binding of O_2_ to hemoglobin and antagonistic action of enzymes, to multiple phosphorylation cascades, bistability in cell cycle checkpoints, or circadian rhythms. This review offers a guide to understand how network switches can be described mathematically and highlights the crucial role of network switches in the generation of self-sustained oscillations.

### Design principles of circadian oscillators

Circadian rhythms have a particularly long and well-controlled period of about 24 h. Mathematical theory predicts that such periods require a delay of at least 6 h (see Box 4). The underlying molecular mechanisms that can contribute to such long delays have been debated for decades (86, 87, 89, 90, 91, 92). But it is becoming clear that, at least in mammals and fungi, multiple phosphorylations of the IDPs PER2 and FRQ are likely candidates to provide the necessary delays (90, 91).

In most organisms, the negative circuits required to generate self-sustained oscillations emerge as a result of TTFLs. There are, however, additional mechanisms that have been shown to contribute to circadian rhythmicity in the absence of transcription: based on phosphorylation reactions and on redox reactions.

Clocks in cyanobacteria have been reconstituted *in vitro* in test tubes (152) and have little in common with TTFLs (153). The core mechanism is based on KaiA-assisted phosphorylation of KaiC tetramers, a process that happens within 12 h. Later in the cycle, KaiA–KaiB–KaiC complexes get dephosphorylated and disintegrate. These steps contain some of the features that have been discussed over this review, including cooperativity, multiple phosphorylations, complex formation, and sequestration (for a complete review, see (112)). Although the negative feedback loop is not immediately evident, large scale simulations (63) have revealed that the sequestration of KaiA in the fully phosphorylated complex constitutes the required negative feedback. More detailed quantitative models (154) as well as crystal structures of the involved proteins (112, 155) confirm early mathematical predictions from 2007 (63).

In addition, an interesting study by O'Neill and Reddy (156) showed that circadian clocks also run in mammalian erythrocytes. These cells are devoid of nuclei and thus cannot perform rhythms of transcription. The mechanism generating circadian rhythmicity was found to be based on oxidation/reduction reactions that happen in an antioxidant enzyme, peroxiredoxin, that is interestingly conserved across all kingdoms of life (157). Mathematical modeling has helped in deciphering what are the design principles of redox circadian oscillations (158): a series of fast oxidation reactions (which could contribute to a switch-like input-output relation) followed by a delayed negative feedback loop where either peroxiredoxins get degraded by the proteasome (159) after post-translational modifications or the proteins get reduced back to the active peroxiredoxin state (160).

Network switches involve nonlinearities that can arise due to cooperativity and complex formations. It is well known from oscillator theory that nonlinearities generate ‘harmonics’, that is, rhythms with half or even a third of the original period (161). Interestingly, such 12 h and 8 h harmonics have been found experimentally in transcriptional profiles of the mammalian (162, 163) and the *Neurospora* clock (164). Thus, switch-like nonlinearities are not only necessary for limit cycle generation but also provide additional rhythmicities. The physiological relevance of these harmonics are still debated (165) and might even link circadian rhythms to tidal oscillations (161, 166, 167).

It is important to note that single cells are inherently noisy. Although the mean mRNA levels of core clock genes oscillate in a circadian manner over the 24 h cycle, mRNA counts show considerable spread between cells (101). Part of this variability might be attributed to heterogeneity in cell size, transcriptional bursting, or external sources of noise. Consequently, deterministic ODE or DDE models have clear limitations when describing transcription-based circuits at the single cell level. However, they do offer a rather simple perspective on the underlying processes and offer valuable insights into the system dynamics. Despite the increasing efforts to understand how the mRNA distributions of core clock circadian genes evolve over the circadian cycle while considering intrinsic and extrinsic sources of noise, it remains to be understood how low and noisy numbers of mRNA counts can generate relatively stable protein oscillations at the single cell level, as illustrated by the long term recording of mouse single fibroblasts by Leise *et al*. (93). It is plausible that network switches may help explain some of these questions.

### Concluding remarks

Throughout this review, we have revisited what biological mechanisms can give rise to network switches and how these can be described mathematically. We have discussed their role in governing cell fate and pathway activation and how they can contribute to rhythm generation of different timescales. An understanding of network switches can thus provide insights into the mechanisms through which clocks coordinate with the environment and into the intricate relationship by which clocks and switches control health and disease. Ultimately, the design principles of network switches and cellular clocks might provide a rich resource to allow their regulation in the context of cellular function and to pave the way for the future generations of medicine, including circadian medicine.

## Conflict of interest

The authors declare that they have no conflicts of interest with the contents of this article.
